# Application of the procedural consolidation concept to surgical treatment of children with epidermolysis bullosa: a retrospective analysis

**DOI:** 10.3325/cmj.2011.52.520

**Published:** 2011-08

**Authors:** Maja Karaman Ilić, Josipa Kern, Irena Babić, Diana Šimić, Antun Kljenak, Višnja Majerić Kogler

**Affiliations:** 1 Department of Anesthesiology, Resuscitation, and Intensive Care, University Hospital for Lung Diseases Zagreb, Croatia; 2 Department of Medical Statistics, Epidemiology and Medical Informatics, Andrija Štampar School of Public Health, University of Zagreb School of Medicine, Croatia; 3 Department of Surgery Children's Hospital Zagreb, Croatia; 4 Faculty of Organization and Informatics, Varaždin, Croatia; 5 Department of Surgery Children's Hospital Zagreb, Croatia; 6 University of Zagreb, School of Medicine, Croatia

## Abstract

**Aim:**

To assess the efficacy of the procedural consolidation concept (PCC) at reducing the number of sessions of general anesthesia necessary for treating children with epidermolysis bullosa (EB).

**Methods:**

We examined the records of children treated at Children's Hospital of Zagreb between April 1999 and December 2007. Children treated before the introduction of PCC in January 2005 (n = 39) and after (n = 48) were analyzed in order to determine the effect of PCC on the occurrence of complications, days of hospitalization, and number of hospitalizations.

**Results:**

During the study period, 53 patients underwent 220 sessions of general anesthesia for a total of 743 surgical interventions per session. Before the introduction of PCC (n = 39 patients, 83 sessions), the median number of interventions per session was 2 (range 1-5), and after the introduction of PCC (n = 48 patients, 137 sessions) it was 4 (range 3-7, *P* < 0.001). After the introduction of PCC, the median number of complications per anesthesia session increased from 2 (range 0-10) to 3 (range 0-10) (*P* = 0.027), but the median number of complications per surgical procedure decreased from 1 (range 0-10) to 0.6 (range 0-2.5) (*P* < 0.001). PCC lengthened each anesthesia session from a median of 65 minutes (range 35-655) to 95 minutes (range 50-405), (*P* < 0.001). Total length of hospitalization was similar before (median 1, range 1-4) and after (median 1, range 1-3) introduction of PCC (*P* = 0.169). The number of hospitalization days per procedure was 3 times lower after the introduction of PCC (median 0.3, range 0.2-3) than before (median 1, range 0.75-1.7) (*P* < 0.001).

**Conclusion:**

PCC should be considered an option in the surgical treatment of children with EB.

Epidermolysis bullosa (EB) refers to a group of rare genetic disorders characterized by cutaneous and extracutaneous fragility, blistering, and subsequent scaring even after a mild mechanical stress (1). The hallmark feature of inherited EB is mechanical fragility (2). The illness is chronic, degenerative, difficult to treat, and lethal. Although the most common symptom of EB is bullae of skin epithelium, blisters may arise on virtually any mucosal surface (3). Bullae usually heal, leaving atrophic scars. Digital fusion following scar formation is common, rendering the hands useless (3).

Traditionally, EB is divided into three major types based on phenotype, mode of inheritance, and genotype (4): EB simplex, junctional EB, and dystrophic EB. More recently, a fourth form, hemidesmosomal EB, was introduced to describe EB in which blisters emerge in the dermal-epidermal circuit. Dystrophic EB is probably the most common form of surgically treated EB (5). Each major EB type has subtypes (4,6,7); two major subtypes of dystrophic EB are dominant dystrophic EB and recessive dystrophic EB. The overall prevalence for inherited EB in the American population is 8.22 per million (8). Prevalence of EB simplex, junctional EB, dominant dystrophic EB, and recessive dystrophic EB in the United States of America (USA) is 10.75, 2.04, 2.86, and 2.04 per million, respectively (8). Determining accurate prevalence and incidence data on inherited EB in the USA became possible only after the national EB registry was established in 1986 (9-11). A Croatian national EB registry does not yet exist. According to a study in 1990 on a limited number of EB patients, the prevalence of the Hallopeau-Siemens subtype of recessive dystrophic EB in Croatia was estimated at 9.6 people per million (12).

EB has become recognized as a multisystem disorder that poses a number of pre-, peri-, and postoperative challenges (13). Scars, strictures, and stenoses are some of the problems that require surgical treatment (14). In addition, EB affects the oral-gastrointestinal system, creating the need for operative treatment and significantly affecting perioperative care (13).

Patients with EB are a high-risk group for anesthesiology because of malnutrition, anemia, hypoproteinemia, dilated cardiomyopathy, and limited mouth opening (15,16).

A multidisciplinary team approach to EB treatment is recommended. Preoperative preparation must be individualized, with special attention given to the potential anesthetic problems. When procedures under anesthesia are planned, it is best to coordinate as many surgical procedures as possible to avoid repeated anesthesia (17).

Surgical care for EB patients at the Children's Hospital of Zagreb was for many years quite basic and was not codified into a uniform and standard procedure. In January 2005, the hospital adopted a team approach to treating EB, which led to implementation of a procedural consolidation concept (PCC).The standard approach of repeated sessions of anesthesia with no more than two surgical procedures performed each time (18-20) was replaced with PCC, which implied multiple surgical approaches and procedures during the same session of anesthesia. PCC is based on multidisciplinary team-based treatment planning aimed at reducing the number of times that patients with EB are subjected to general anesthesia. In this study, we analyzed whether PCC was successful at reducing the number of anesthesia sessions without increasing complications or length of hospitalization.

## Methods

This is a retrospective study of medical records of all patients with EB who were surgically treated under general anesthesia in Children's Hospital of Zagreb between April 1999 and December 2007. The study was approved by the Institutional Review Board of Children's Hospital of Zagreb. Written informed consent was obtained from patients, their parents, or their legal guardians prior to each anesthesia session.

All patients scheduled for surgery were reviewed by an EB team composed of a pediatrician, dermatologist, anesthesiologist, and two surgeons. Based on clinical examination by the multidisciplinary team and laboratory findings, individual plans for surgical treatment were created for patients with EB. Patients were hospitalized on the day of surgery.

 A total of 220 anesthesia sessions were divided into two groups, those performed before (n = 83 sessions, 39 patients) and after (n = 137 sessions, 48 patients) the introduction of PCC. The groups of patients treated before and after the introduction of PCC were compared in terms of observed complications per general anesthesia session, per 10 minutes of anesthesia, total length of hospital stay, and number of hospitalizations. All perioperative complications were defined as short-term complications the occurrence of which did not affect duration of hospital stay. Follow-up period was during the hospital stay.

### Premedication and anesthesia

Oral premedication was used in 41 (19%) of 220 sessions of general anesthesia. Midasolam was administered in syrup form. Intravenous (IV) access before induction in anesthesia was achieved in only 36 (16%) of 220 sessions, 21 of which were in children who had been premedicated. In all cases, induction in anesthesia was by inhalation. Until 2004, the inhalational anesthetic was Halothane (Halotan); from 2004 onwards, it was Sevofluran (Sevoran). Of the total of 220 sessions of anesthesia, 127 (58%) were carried out entirely with a “face” mask, while in the remaining 93 (42%) there were indications for endotracheal tube insertion. Most intubations (62 of 93, 67%) were performed after introduction of PCC. A fiber-optic bronchoscope (Olympus BF-3C-20; Olympus Europa GmbH, Hamburg, Germany) was used for intubation of infants and small children. For children over 10 years, an Olympus BF-P-20-D was used. The patients did not consent to the use of regional anesthesia.

The neuromuscular relaxant rocuronium and the opioid analgesic fentanyl were used to ensure good intubation and calmness during the surgery. Pulse oximeter oxygen saturation, electrocardiography, non-invasive blood pressure, blood loss, and body temperature were monitored during surgery. The face mask and skin exposed to friction were lubricated with Vaseline. Electrocardiogram electrodes were kept in place with bandages, and Mepitel (Mölnlycke Healthcare AB, Göteborg, Sweden) and soft gauze bandages were used to care for wounds and to secure the IV line.

### Postoperative pain control

For postoperative pain monitoring a visual-analogue scale (VAS) was used. Pain in infants was assessed in most cases by the mother using a VAS. In two cases, the infant CRIES scoring system (21) was combined with mother's assessment of the infant's discomfort using the VAS.

To control pain during the early postoperative period, a multimodal approach was used. Non-steroidal anti-inflammatory drugs were administered with tramadol through continuous IV infusion. Suppositories were not used. If needed, sedatives were also administered. Patients showed normal respiration and reported feeling no pain. For prevention of postoperative nausea and vomiting (PONV), metoclopramide or dexamethasone was used.

### Statistical methods

Descriptive statistics, including median and range for the number of surgical procedures, were used. Frequencies of complications were presented as absolute values and percentages. For not normal distributions of variables, the Kruskal-Wallis test was used to test the significance of pre- and post-intervention outcomes.

## Results

The sample consisted of 53 (31 male and 22 female) children with EB from 7 different countries who underwent a total of 220 sessions of general anesthesia. Fifty-one of the 53 children underwent several sessions of anesthesia several times in a relatively short period. Eleven children (21%) had EB simplex, 3 (6%) junctional EB, and 39 (73%) dystrophic EB.

The median age at the first surgery was 10 years (range 1-19). Across all surgeries, the median age was 13 years (range 1-23). The median number of anesthesia administrations per patient was 3 (range 1-14).

Fifteen different surgical procedures were carried out 743 times (Table 1). Between April 1999 and December 2004, 150 surgical procedures were performed during 83 sessions of general anesthesia on a total of 39 patients. After the introduction of PCC in January 2005, 593 surgical procedures were performed during 137 sessions of general anesthesia on 48 patients. The median number of procedures before PCC was introduced was 2 (range 1-5), compared with 4 (range 3-7) afterwards (*P* < 0.001). Over the entire study period, the median number of procedures carried out during a single session of anesthesia was 3 (range 1-7).

**Table 1:** Type of surgical procedures performed before and after introduction of procedural consolidation concept (PCC)

Type of surgical procedure	Total number of procedures performed	Performed before PCC, No. (% of total)	Performed after PCC, No. (% of total)
Repair of syndactyly	39	14 (36)	25 (64)
Dilatation of esophageal strictures	42	11 (26)	31 (74)
Tooth repair	20	4 (20)	16 (80)
Tumor excision	10	1 (10)	9 (90)
Circumcision	11	2 (18)	9 (82)
PEG implantation*	4	1 (25)	3 (75)
Plastic reconstructive surgery	112	25 (22)	87 (78)
Body mapping	133	19 (14)	114 (86)
Formation of hand mold	23	9 (39)	14 (61)
Change of dressing	149	27 (18)	122 (82)
Wound tending	147	30 (20)	117 (80)
Biopsy of suspect skin changes	10	1 (10)	9 (90)
Contracture release	24	4 (17)	20 (83)
Ear/neck reconstructive surgery	16	2 (13)	14 (87)
Emergency	3	0 (0)	3 (100)

*PEG – percutaneous endoscopic gastrostomy.

**Figure 1:**
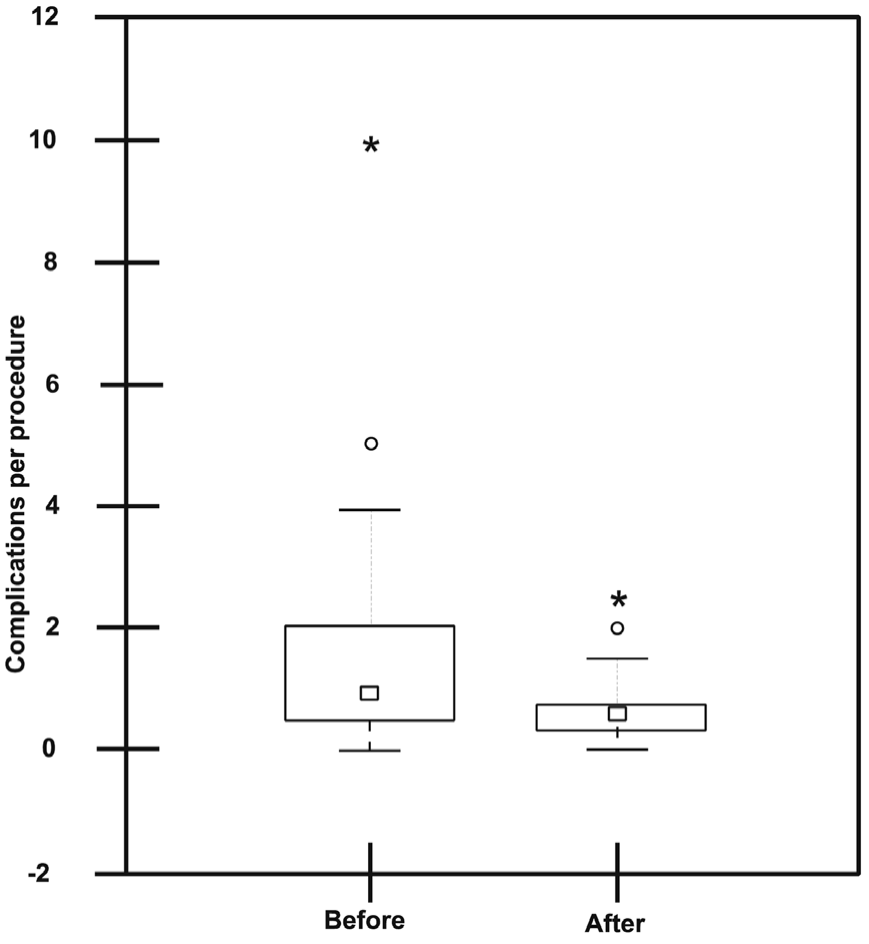
Distribution of the number of complications per procedure carried out during a single session of general anesthesia before and after the introduction of procedural consolidation concept. Small square – median, box – 25%-75% interval, whiskers – non-outlier range, circles – outliers, asterisk – extremes.

Development and frequency of 19 different complications were noted (Table 2). Complications occurred in all 53 patients. Over the entire period, the median number of complications per session of anesthesia was 2 (range 0-10). The median number of complications per session of anesthesia increased after the introduction of PCC from 2 (range 0-10) to 3 (range 0-10) (*P* = 0.027). In contrast, the median number of complications per surgical procedure decreased from 1 (range 0-10) to 0.6 (range 0-2.5) after the introduction of PCC (*P* < 0.001) (Figure 1). The number of complications per patient was significantly lower before (median 2, range 1-20) than after (median 3, range 1-26) the introduction of PCC (*P* < 0.001).

**Table 2:** Complications relative to the number of anesthesia sessions and surgical procedures before and after the introduction of procedural consolidation concept (PCC)*

Complication type	Overall			Before PCC			After PPC		
	total number	% per No. of anesthesia sessions (n = 220)	% per No. of surgical procedure (n = 743)	total number	% per No. of anesthesia sessions (n = 83)	% per No. of surgical procedures (n = 150)	total number	% per No. of anesthesia sessions (n = 137)	% per No. of surgical procedures (n = 593)
Drop in RBC count	63	29	8	21	25	14	42	30	7
New head and neck bullae	50	23	7	16	19	11	34	24	6
Oropharyngeal bullae	59	27	8	17	20	11	42	31	9
New skin injuries	14	6	2	7	8	5	7	5	1
PONV	58	26	8	23	28	15	35	26	6
Pain requiring treatment	104	47	14	31	37	21	73	53	12
Teeth damaged	12	5	2	4	5	3	8	6	1
Corneal erosions	4	2	1	1	1	1	3	2	1
SpO_2_≤90%	43	20	6	13	16	9	30	22	5
Multiple intubation attempts	20	9	3	7	8	5	13	9	2
Laryngospasm	6	3	1	1	1	1	5	4	1
Aspiration	3	1	0.4	0	0	0	3	2	1
Arrythmia	32	15	4	11	13	7	21	15	4
Hypotension	54	25	7	21	25	14	33	24	6
Delay awakening	6	3	1	2	2	1	4	3	1
Mechanical ventilation	3	1	0.4	1	1	1	2	1	0.3
Allergic reaction	3	1	0.4	1	1	1	2	1	0.3
Hypothermia	26	35	3	9	11	6	17	12	3
Swelling of IV line insertion	48	22	6	19	23	13	29	21	5

*Abbreviations: RBC – red blood cells; PONV – post-operative nausea and vomiting; SpO_2_ – pulse oxymeter oxygen saturation; IV – intravenous.

PCC lengthened each anesthesia session from 65 minutes (range 35-655) to 95 minutes (range 50-405) (*P* < 0.001). The median number of surgical procedures per 10 minutes of anesthesia increased from 0.3 (range 0.0-0.6) to 0.4 (range 0.1-0.9) (*P* < 0.001), without a change in the median number of complications per 10 minutes: 0.3 (range 0-1.3) vs 0.3 (range 0-0.9) (*P* = 0.56).

**Figure 2:**
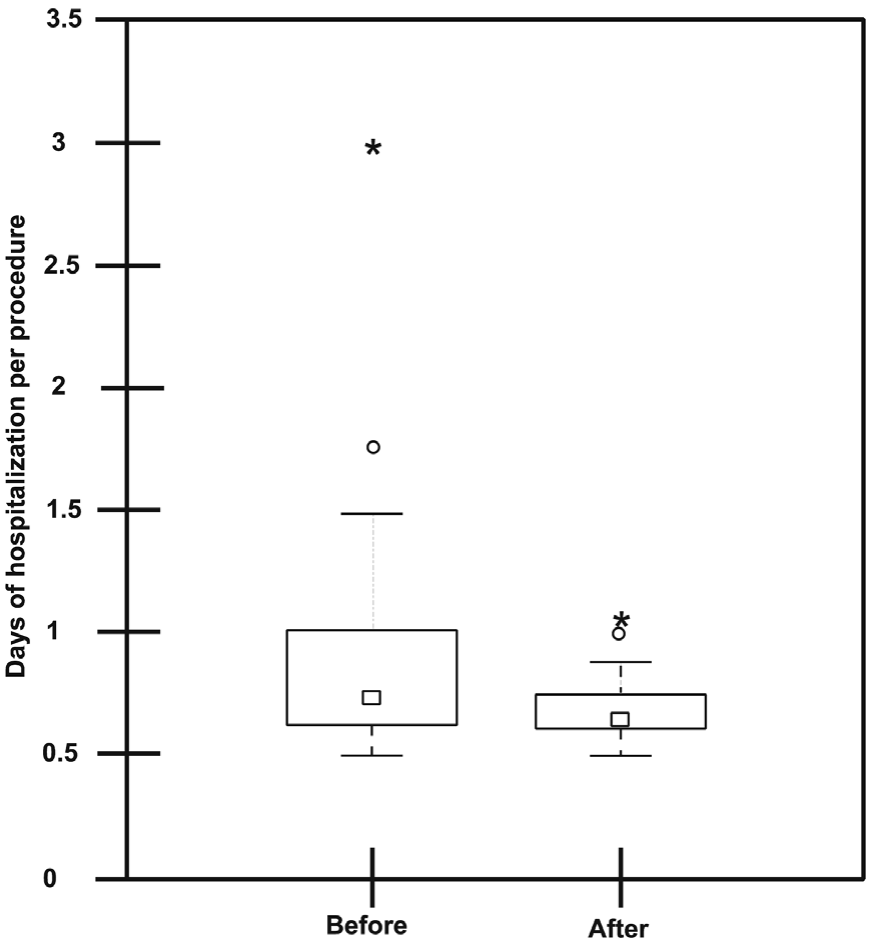
Distribution of days of hospitalization per procedure carried out during a single session of general anesthesia before and after introduction of procedural consolidation concept. Small square – median, box – 25%-75% interval, whiskers – non-outlier range, circles – outliers, asterisk – extremes.

Over the entire study period, the median number of hospitalization days was 1 (range 1-4). The number of hospitalization days per session of anesthesia was not significantly different before (median 1, range 1-4) and after (median 1, range 1-3) the introduction of PCC (*P* = 0.169). In contrast, the number of hospitalization days per surgical procedure was significantly higher before (median 1, range 0.75-1.75) than after (median 0.3, range 0.2-3) the introduction of PCC (*P* < 0.001) (Figure 2).

## Discussion

This study was designed to investigate the impact of carrying out multiple surgical procedures during a single session of general anesthesia in the treatment of children with EB. Our results show that after the introduction of PCC, anesthesia sessions became longer and the number of surgical procedures during one session increased. Although the absolute number of complications increased, the rate of complications per 10 minutes of anesthesia did not change. The overall length of hospital stay did not change, but the number of hospitalizations decreased significantly.

Although most surgical procedures on adult patients can be performed under local or regional anesthesia, other criteria apply to children with EB. The disease makes children more vulnerable and less tolerant, so even minor and trivial operations such as dressing changes, body mapping, and formation of hand molds are performed under general anesthesia.

During the study period, 15 different surgical procedures were performed and the occurrence of 19 complications was recorded. The frequency and number of complications in our study differ from previously published results (18-20,22). Iohom et al (1) reported their 20-year experience with anesthesia for children with EB. Fifty-six anesthesia sessions (54 general and 2 local) were performed for 58 procedures in 10 children. Two complications related to anesthesia occurred 10 times (PONV, 7; new bullae, 3). We observed a far higher rate of complications: complications arose in 209 of 220 anesthesia sessions, despite the application of preventive measures. This can be explained by the fact that development of typical complications for patients with EB (new head and neck bullae and/or new oropharyngeal bullae) were reported together with adverse events associated with anesthesia (intubation, reaction to medications used in the anesthesia, irregular heartbeat, changes in blood pressure, pain and nausea, and vomiting) and with complications related to surgery (drop in red blood cells, hypothermia).

Patients with EB are most vulnerable during the intubation period and it is the time when most complications appear. Our study showed that after the introduction of PCC there was an increase in the number of surgical procedures involving the oral cavity (dilatation of esophageal strictures, teeth repair) for which patients should be intubated (19,1,23). Surgeon and anesthesiologist share the same workspace which increases the chance of complications. This may explain the higher number of complications per anesthesia session after the introduction of PCC. Higher number of complications however did not influence duration of hospital stay.

Use of a laryngeal mask as an alternative to intubation was not considered for several reasons: it would reduce the already small workspace available for delicate procedures such as esophageal dilatation and tooth repair; most anesthesia sessions for syndactyly repair lasted longer than two hours, which is a contraindication for laryngeal mask; and the anesthesiologist on our team was well-trained in fiber optic intubation.

Although introduction of PCC led to a greater number of complications, this increase did not significantly prolong hospital stay. At the same time, PCC reduced the number of hospitalizations due to the smaller number of sessions of general anesthesia. The 593 surgical procedures that were carried out in the post-PCC group during 137 sessions of general anesthesia and 137 hospitalizations would have required, in the pre-PCC approach, 2.4-fold more hospitalizations, with the associated risks and costs. A reduction in the number of hospitalizations, without any decrease in health services, translates into improved quality of life for children with EB, since it means less stress and distortion of established routines.

Although this study used a relatively large sample, there are some important limitations. The main ones are the retrospective study design and insufficient data about the influence of patient age, co-morbidity, and the severity of complications. In addition, we examined only short-term perioperative complications. Patients were discharged from hospital when these complications were considered no longer life-threatening to patients. Although we did not perform longer-term follow-up, we were nevertheless able to show satisfactory results for standard EB findings, such as anemia and positive skin microbiological culture. Our patients were discharged with the recommendation that they continue outpatient supervision and treatment with a competent pediatrician and dermatologist. During the study period, discharged patients did not report any problems related to recently performed surgical procedures that would have required re-hospitalization.

Our results suggest that PCC is effective at reducing the exposure of patients with EB to the risks of general anesthesia. This approach requires the involvement of multidisciplinary teams to plan, prepare, and coordinate treatment for each patient.
